# Diode laser photocoagulation of intraoral (and perioral) venous malformations: Cases series

**DOI:** 10.1016/j.ijscr.2021.106436

**Published:** 2021-09-28

**Authors:** Dounia Sarfi, Salma Adnane, Sofia Haitami, Ihsane Ben Yahya

**Affiliations:** Oral Surgery, Dental Consultation and Treatment Center, Ibn Rochd University Hospital Center, Casablanca, Morocco, BP: 9157

**Keywords:** Vascular abnormality, Venous malformation, Photocoagulation, Diode laser

## Abstract

Vascular anomalies are subdivided into vascular tumors (hemangiomas) and vascular malformations. They are frequently located in the head, neck, and oral cavity. They are common complaints reported in patients seeking treatment for aesthetic or functional issues.

However, recent advances in the diagnosis and management of these lesions are improving treatment strategies. This review provides both basic and up-to-date knowledge on the most common vascular anomalies encountered by practitioners.

Due to the wide variability of treatment options which often generates debate, this paper work aims to provide a comprehensive approach of these lesions based upon current concepts and practical clinical experience.

Our article is about 4 patients who had consulted for one or several purplish, elevated, well limited and soft lesion. These lesions was not painful, but worrying for patients. Therapies for VAs continue to generate a dilemma for oral surgeons. Several treatment options were reported, including conventional surgery with or without adjunctive preoperative embolization, and drug therapies, such corticosteroids, intralesional injection of corticosteroids and intralesional injections of sclerosing agents. All of these therapeutic approaches carry a high risk of severe side effects such as scars, pain, and bleeding [7].

Nowadays, advances in the use of lasers have allowed doctors an effective treatment with minimal side effects [9].

All our cases described in this article were done by Pr Haitami, using the 980 nm Diode laser, and a complete healing was observed in about 8 months at the most. The laser is therefore a great help in the management of this type of lesion.

## Highlights

• The last classification and clinical aspects of vascular anomalies
• Contribution of the laser in the management of angiomas
• The use of the 980 nm diode laser to the sclerosis of the venous malformation by intralesional photocoagulation

## 1 Introduction

Vascular anomalies (VAs) arise from blood vessel abnormalities or during endothelial proliferation. Interdisciplinary diagnosis and treatment are of real importance in these cases. The classification separates the vascular anomalies into two major groups. The first group includes tumors associated with a process of endothelial cell proliferation, which appears in childhood, with proliferative changes, followed by an involutional process of unknown etiology, called hemangiomas and the second one, consisting of those vascular malformations (VM) that appear as a result of the altered development and formation of blood vessels, present at the birth, normal endothelial mitotic activity, which grows throughout life with no presence of an involutional process [2]. These malformations can show any combination of capillary, venous, arterial, or lymphatic components, presence or absence of a fistula. All VAs can also be differentiated on the basis of vascular flow, either low or high, that serves as an adjunct indication that can be used to make a correct diagnosis [3]. The occurrence of vascular anomalies in the orofacial area is a common condition. Traditional treatment approach, such as surgery and chemical sclerosis, has been giving way to a few options with a less invasive approach, such as the use of the 980 nm diode laser to induce the sclerosis of the venous malformation by intralesional photocoagulation [1]. It is a renovating technique with less follow-up surgery.

## 2 Methods

The research registry number in accordance with the Declaration of Helsinki is researchregistry6957 (https://www.researchregistry.com/browse-the-registry#home/registrationdetails/60e745f6c3cfb0001f4b239b/).

The prospective study described in our article is a case series, and is unique.

All our patients were treated in a hospital setting, by Pr Haitami (Professor, D.M.D., Specialist in Oral Medicine and Oral Surgery), at the Center for Dental Consultations and Treatments at the CHU Ibn Rochd Hospital in Casablanca.

All of our patients were recruited between 2018 and 2021, and received a follow-up that lasted up to 8 months.

No particular precautions were taken before the intervention.

This case series has been reported in line with the PROCESS Guideline [6].

### 2.1 Case 1

**Fig. 1:**
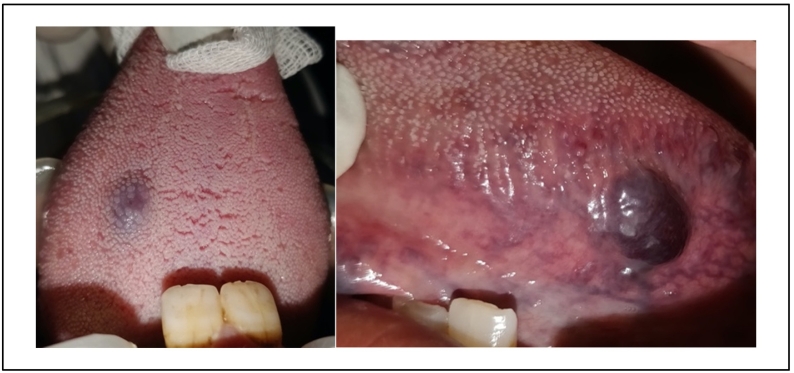
Two dark blue-colored papules: one on the dorsal side of the tongue, and the other on its lateral side. (For interpretation of the references to color in this figure legend, the reader is referred to the web version of this article.)

A 61-year-old woman, with no particular medical history, was referred to our department for an evaluation regarding two oral bluish lesions. She reported that these lesions appeared about 5 months ago. The oral mucosa examination showed two dark blue-colored papules: one on the dorsal side of the tongue and the other on its lateral side. Each lesion was of 1-cm diameter, elevated, and well-limited. A pulsating sensation was perceived during the palpation [Fig. 1].

**Fig. 2:**
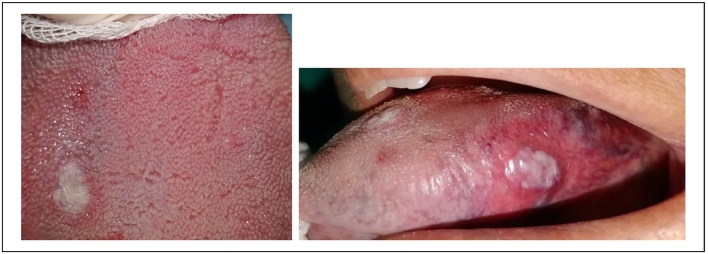
Intraoperative photo of whitening of the lesions.

During palpation, the papule was soft and whitened with applied pressure. The diagnosis of venous malformation was established. A very light anesthesia (Mepivacaine) was performed all around the lesion. We performed a photocoagulation of the lesion using a 980-nm diode laser in noncontact mode. We used a 300-nm fiber in continuous wave, with a power of 1.5 W for 1 min until the lesion whitens [Fig. 2].

**Fig. 3:**
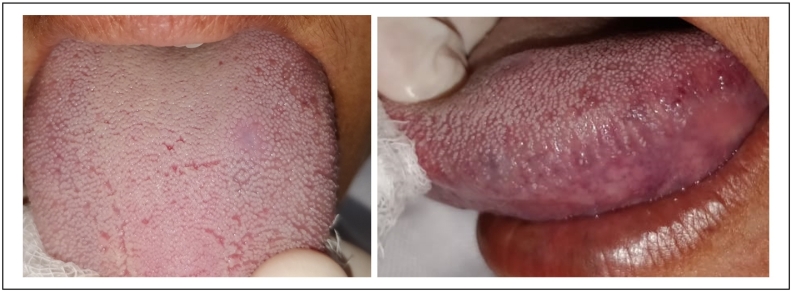
A perfect healing process after 30 days.

One gram of paracetamol every 6 h was prescribed during 3 days. The follow-ups at 10, 20, and 30 days showed a perfect healing process. The patient did not report any post-operatory complications or recurrence [Fig. 3].

### 2.2 Case 2

**Fig. 4:**
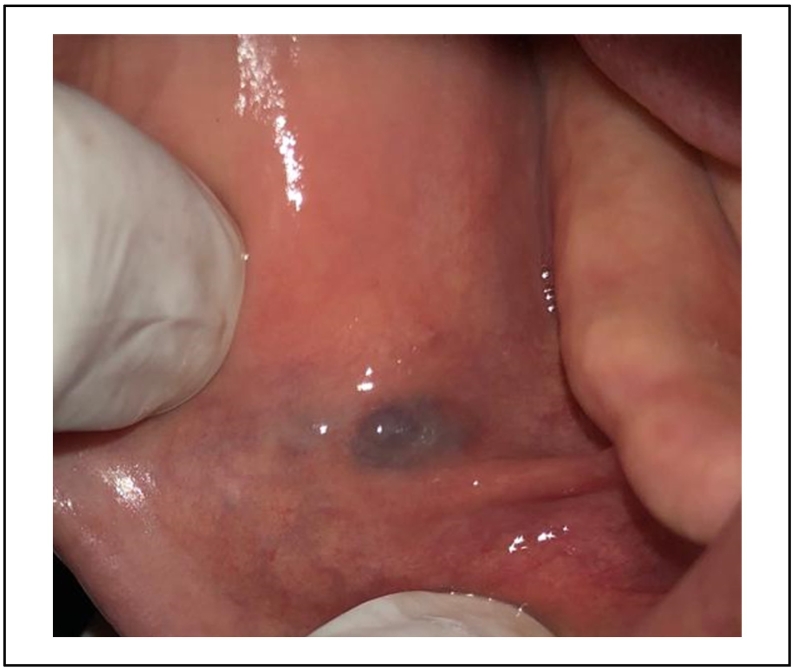
A soft, round purplish, elevated and well-limited lesion.

A 65-year-old female patient, with no particular medical history, was referred to the Department of Oral surgery for diagnosis and treatment of a soft round tumor of 5 mm diameter approximately in her jugal mucosa. The extra-oral exam has shown respected facial symmetry. According to the clinical examination, the lesion was soft, round, purplish, elevated, and well limited [Fig. 4].

A pulsating sensation was perceived during the palpation. The diagnosis of venous malformation was established. The vascular lesion was removed in one session after topical anesthesia. The tumor was irradiated using 4Wpower in noncontact mode. The same diode laser (980 nm) was used, and the fiber had not been activated.

**Fig. 5:**
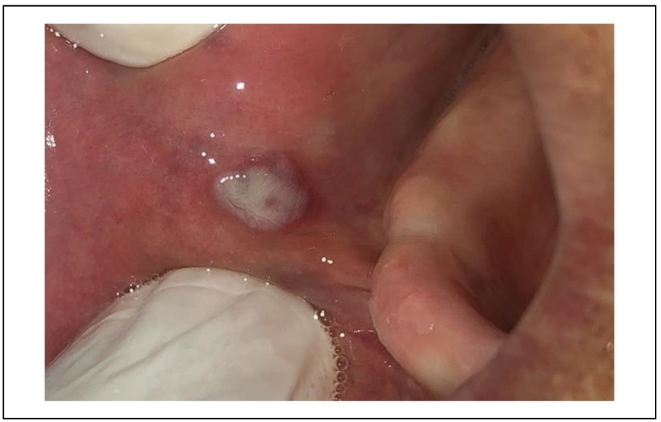
Intraoperative photo of the lesion's bleaching.

We worked 2–3 mms away from the surface of the lesion by sweeping the lesion from the center to the periphery. The lesion started to bleach gradually [Fig. 5]. An ice cube was used for a cooling effect. This effect was able to prevent tissue heating and therefore to control necrosis.

**Fig. 6:**
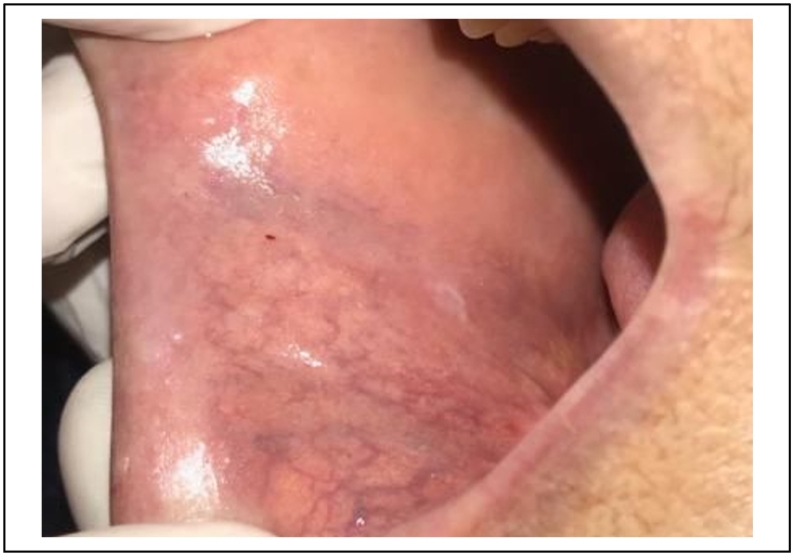
A complete healing after 4 weeks.

Postoperative medication was not mandatory. A superficial layer of fibrin was present after 10 days, and four weeks after surgery the oral mucosa was completely healed [Fig. 6].

### 2.3 Case 3

A 40 -years-old woman, with no particular medical history, was referred to the department of oral surgery for a purplish lesion increasing in volume, which had appeared 1 year ago on the inner side of the upper lip and the vermilion.

**Fig. 7:**
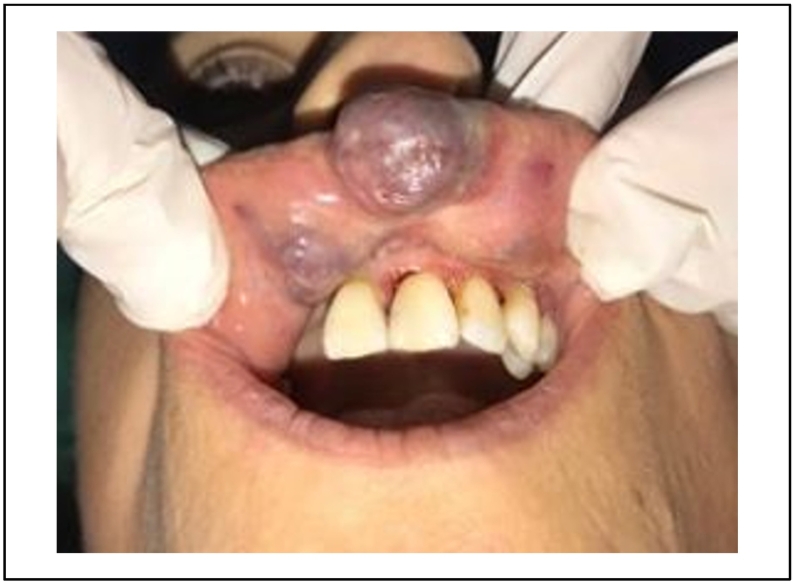
2 purplish lesions with a diameter on the inner side of the upper lip.

The patient's medical and surgical history revealed a trauma to the upper lip during childhood that went unnoticed [Fig. 7].

The extra oral clinical examination revealed a bluish swelling in the upper lip causing significant aesthetic damage.

The intra-oral examination showed two other purplish lesions, well limited to the inner side of the upper lip, slightly indurated, and pulsated during palpation [Fig. 7].

The diagnosis of hemangioma was established.

A 980 nm diode laser was selected for treatment of the lesion in a defocused mode, with an output power of 4 W, in a continuous mode under local anesthesia.

During the first 48 h, the patient reported a slight discomfort because of the swelling.

The patient did not return for the follow-up appointment due to restrictions related to the corona pandemic.

She was seen 8 months later, partial healing was observed.

The residual lesion was coagulated by the same diode laser with the same settings.

After 45 days, clinical healing was observed with no signs of inflammation or infection.

**Fig. 8:**
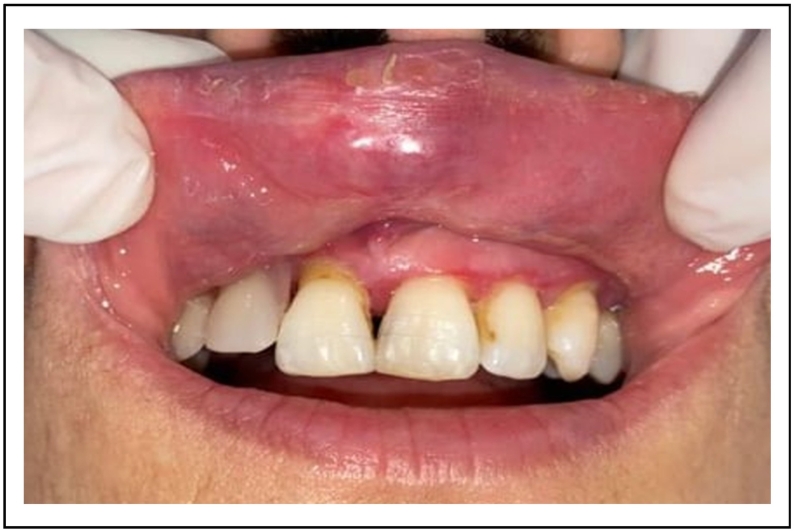
8 months later, complete healing observed on the inner side f the upper lip and persistant lesion of the vermilion.

After 3 months, a full recovery of the area and a reestablishment of the facial aesthetic was observed [Fig. 8].

### 2.4 Case 4

**Fig. 9:**
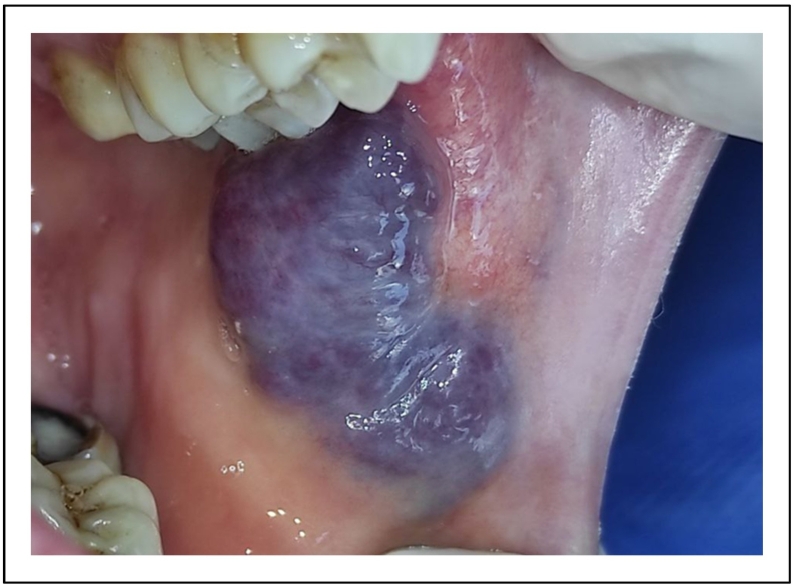
Purplish lesion on the inner side of his left cheek.

A 30 -years-old woman, with no particular medical history, was referred to the department of oral surgery for a purplish lesion increasing in volume, which had appeared a few months ago on the inner side of his left cheek. The extra-oral exam has shown respected facial symmetry. According to the clinical examination, the lesion was soft, purplish, elevated, and well limited [Fig. 9].

A pulsating sensation was perceived during the palpation. The diagnosis of hemangioma was established.

**Fig. 10:**
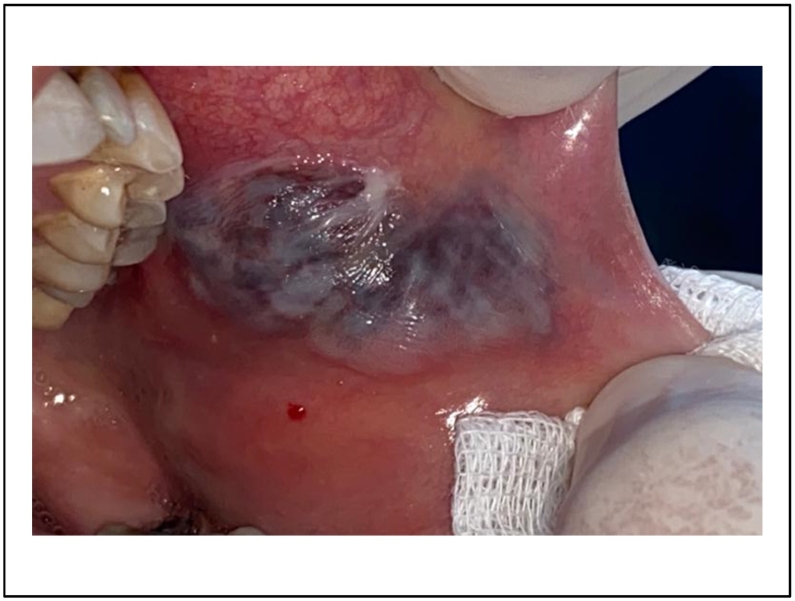
A complete healing after 4 months.

The vascular lesion was removed in one session after topical anesthesia. The tumor was irradiated using 4Wpower in noncontact mode. The same diode laser (980 nm) was used, and the fiber had not been activated. The lesion started to bleach gradually [Fig. 10]. Postoperative medication was not mandatory.

**Fig. 11:**
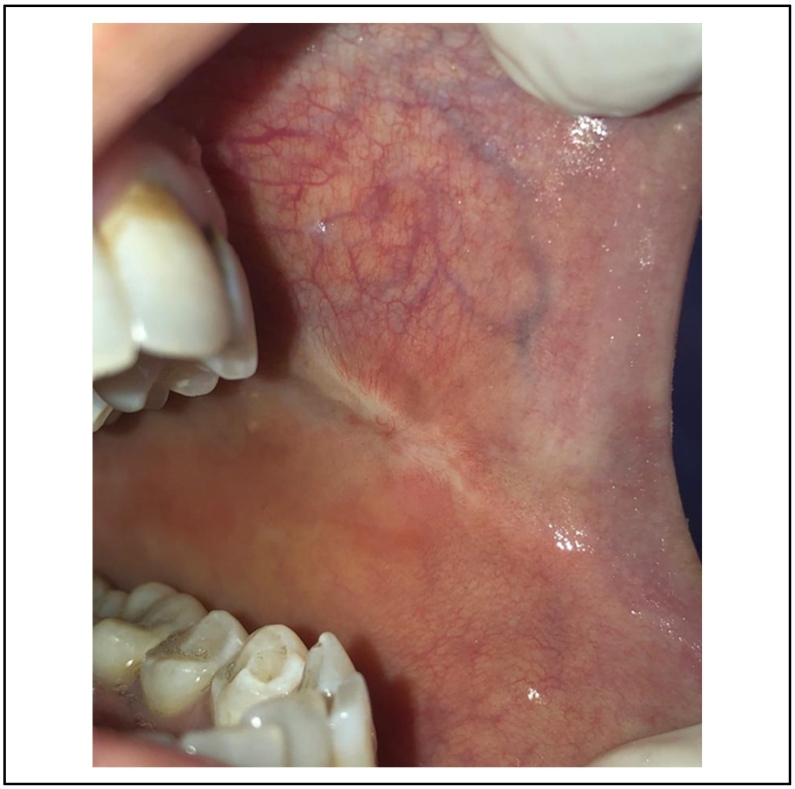
Intraoperative photo of the lesion's bleaching.

4 months after surgery, the oral mucosa was completely healed [Fig. 11].

At her follow-up visit, the patient reported: <Thank you doctor, I did not feel any pain after the operation>.

## 3 Discussion

**Fig. 12:**
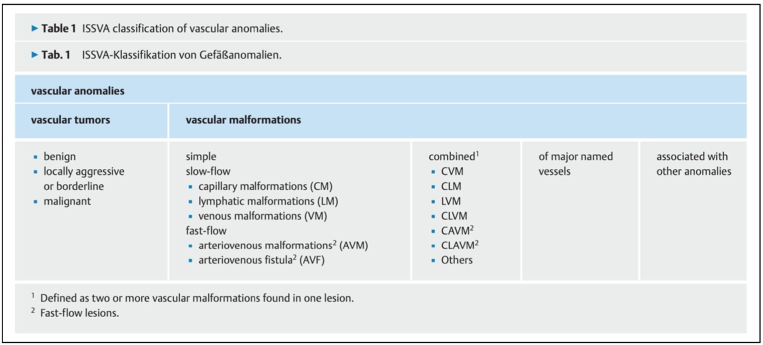
ISSVA classification for vascular anomalies.

The characteristics and differences concerning vascular anomalies (VAs) have been widely discussed and revisited. Recent reanalysis ruled out the latest classification system of the International Society for the Study of Vascular Anomalies (ISSVA), published in May 2018 [Fig. 12].

(Approved at the 20th ISSVA Workshop, Melbourne, April 2014, last revision, May 2018)

The (ISSVA) categorizes vascular anomalies into vascular tumors and vascular malformations. Vascular malformations are further divided into slow-flow (venous, lymphatic, and capillary malformations) and fast-flow malformations (arteriovenous malformations and arteriovenous fistulas).

This interdisciplinary classification has therapeutic implications. Despite its accurateness, it has a clear shortcoming in regard to interdisciplinary communication [4]. Vascular tumors may be subclassified as benign, locally aggressive, borderline, and malignant tumors with multiple patterns of histological and clinical manifestations. The most common representative of vascular tumors are hemangiomas. They are characterized by endothelial cell proliferation and angiogenesis and aren't necessarily present immediately after birth, but may develop in the first few weeks of life and start regressing during puberty or earlier [5]. Infantile hemangiomas are much more frequent (90%) than congenital hemangiomas (less than 2%). Infantile hemangiomas tend to grow rapidly in the first few months after birth before spontaneous regression in early childhood. The characteristic appearance of an infantile hemangioma is the red, raspberry-like skin coloration.

Congenital hemangiomas differ from infantile hemangiomas as they are fully developed at birth and tend to either regress rapidly, partially or do not regress at all [6].

Vascular tumors are comprised of far more entities than hemangiomas. Based on whether they are malignant or benign, they may require more extensive diagnosis and therapeutic evaluation than hemangiomas.

Clinical inspection and patient history are essential for appropriate diagnosis.

Vascular malformations are always present at birth (even if asymptomatic) and never regress spontaneously. They may be quiescent for a long time, before a mechanical or hormonal stimulus results in their growth. With increasing size, vascular malformations can cause pain and functional impairment which may require treatment. Vascular malformations can be classified as “simple” or “combined vascular malformations” as well as “vascular malformations associated with other anomalies” [6].

Furthermore, it is typical of these lesions to only be localized on one side of the body.

As is the situation in our case, where all lesions are unilateral.

The decision for invasive treatment should be made by an interdisciplinary team and is based on individual symptoms and possible complications of the natural course of the disease. The risks of the invasive procedure have to be weighed against the potential benefits [4].

Therapies for VAs continue to generate a dilemma for oral surgeons.

Several treatment options were reported, including conventional surgery with or without adjunctive preoperative embolization, and drug therapies, such corticosteroids, intralesional injection of corticosteroids and intralesional injections of sclerosing agents. All of these therapeutic approaches carry a high risk of severe side effects such as scars, pain, and bleeding [7].

In his article, on the treatment of hemorrhage after dental extraction, related to a capillary hemangioma, Ghanem A et al., described the management by the conventional method [10].

Several materials and embolization protocols are used [10].

Longacre et al. used silicone balls impregnated with barium or tantalum [11].

For a better stability, we can have recourse to the injection of cyanoacrylate of n-butyl [12]. Unlike gel foam, which is used as a temporary means of blocking vessels, polyvinyl alcohol, (which is used usually in embolization), causes a permanent obstruction [13], [10].

According to McHeik and al., intralesional injection of corticosteroids may induce an ulceration of the lesion [8].

Nowadays, advances in the use of lasers have allowed doctors an effective treatment with minimal side effects [9]. Laser- based treatments have multiple benefits: on the one hand, it is conservative, with no bleeding risk, and a reduced necessity of anesthesia, faster healing, more precise cutting, and less postoperative discomfort due to the biostimulative effect. On the other hand, the procedure has no need for sutures, which is superficial with a bloodless operative field and a relative facility and speed of execution [7].

That is why all our patients have been treated by laser.

For effective treatment, the laser must penetrate deep into the target vessel. Additionally, the exposure needs to be long enough to cause sufficient coagulation of these vessels [9].

The interaction between laser and tissue is due to the energy absorbed by tissues. For correct treatment of a lesion with high blood content, it is necessary to choose a laser emitting in a wavelength well absorbed by hemoglobin. KTP, diode, Nd:YAG, and CO2 are the most effective wavelengths in these cases [10] [7].

The 4 cases previously described were treated with A 980 nm diode laser.

Moreover, it is necessary to evaluate many further parameters such as: pulse duration, spot size, and energy density, thus for a correct laser treatment. Shorter pulse durations are preferable for small diameter vessels while longer pulse durations must be employed in larger diameter ones. The spot size selection should be based according to the depth and the size of the lesion [11] Finally, the selection of the energy density should be based on the color of the lesion, purple and bluish lesions absorb laser energy more than pink or red ones requiring for this reason lower fluencies [11].

During their experience, Umberto, Romeo, and al affirm that lasers are the gold standard in the treatment of benign oral vascular lesions of the oral cavity with venous flow [7].

Three different laser approaches were examined in this study: The excisional biopsy(EB), the sole to permit a histological diagnosis of the lesion, must be reserved to vascular lesions suspected to be malignant neoplasms. Among the laser techniques, transmucosal thermocoagulation (TMT) seems to be the most reliable and advantageous.

Indeed, this is the technique that has been used for all previously described cases, in(TMT), the optical fiber is not in contact with the lesion, which makes it safe even in patients affected by systemic diseases such as coagulation problems or under anticoagulant therapy. This method also gives good functional and aesthetic results. Intralesional photocoagulation (ILP) is often used for wide and deeper lesions that cannot be treated with TMT. ILP is characterized by a higher risk of bleeding due to fiber penetration into the lesion, but it can be overall considered a safe technique.

It is possible to assess that the laser is safe and effective, and in many cases it represents the go-on technique permitting results usually unattainable with conventional treatments [7]. Among all lasers that have proven surgical capabilities (e.g., CO2,Nd:YAG, KTP, diode), the diode laser is currently the most widely used for surgical excision of proliferating benign and malignant lesions in the oral cavity [12] .This approach avoids per operative bleeding and reduces the frequency of postsurgical complications [13] which also happened in our cases. Pain, hemorrhaging, scarring, and swelling were not observed in our cases and complete healing was noticed in approximately 8 months at most.

In our case, we found that the ulceration paired up with the healing process was less present in the first patient (1.5 W) compared to the other ones (4 W).

Furthermore, the use of diode lasers either minimizes or completely avoids thermal damage and morpho-structural changes of irradiated tissues [12].

According to several histological studies, the type of diode laser interaction depends on both the type of vascular malformation and the depth of infiltration. Variable absorption capabilities in the underlying tissues are noticed for different vascular malformations, thus highlighting the need for intralesional lasers to treat deep lesions [12], [14].

A study conducted by Álvarez-Camino and al, consisted of the introduction of an activated optical fiber via the catheter lumen, until the active tip is positioned within the venous malformation, keeping it accessible to the lighting guide of the laser unit. The laser is radially applied in the deeper areas of the lesion, while the laser fiber is progressively moved upward to the entrance area of the fiber. Subsequently, the optical fiber is removed. This study has shown promising results [1].

The diode laser light turns into heat when absorbed by hemoglobin, resulting in coagulation. The penetration depth of the 810 nm wavelength appears to be lower than in the Nd:YAG one, making it more effective in the coagulation of superficial and interstitial lesions [5]. Histological studies conducted on the vein walls on which a diode laser session was applied revealed coagulative necrosis causing tissue distortion not in the inner lining of the vessel only, but in the outer layers of the vascular wall as well [15].

The intralesional fiber of the diode laser aims to cause the endothelial injury of the affected vessel. Not only is it induced by the thermal action caused by laser light, but also the secondary thermal effect caused by the bubbles associated with the absorption of light energy by hemoglobin [16].

In conclusion, vascular venous anomalies of small dimensions can easily be treated with diode laser photocoagulation (regardless of the wavelength). It has been observed that most hemangiomas stop growing after laser therapy, and that the rate of adverse reactions is very low which was observed in our case. No sign of recurrence was observed either [17].

## 4 Conclusion

Despite its accurateness, there has been up to now a lack of familiarity with this interdisciplinary classification system. Furthermore, when asked how to manage peripheral vascular malformations, we are faced with a large number of studies and case series with low levels of evidence and mixed results, which in the face of individual varying patient presentations makes it nearly impossible to decide what the optimal treatment strategy for each patient is [4] Other lasers can achieve better success rates, because of their better absorption of hemoglobin. These include the Argon laser and Nd: YAG KTP. Both emit a green light that matches the peak of maximum absorption of hemoglobin.

Nevertheless, several questions regarding laser treatment for VeMs remain unresolved, especially those related to challenging differential diagnosis, the sites involved, depth of localization, and lack of standardized treatment protocols [14].

## Funding

The authors declared that this study has received no financial support.

## Ethical approval

Written informed consent was obtained from the patient for publication of this case report and accompanying images. A copy of the written consent is available for review by the Editor-in-Chief of this journal on request.

## Provenance and peer review

Not commissioned, externally peer-reviewed.

## Consent

Written informed consent was obtained from the patient for publication of this case report and accompanying images. A copy of the written consent is available for review by the Editor-in-Chief of this journal on request.

## Registration of research studies

1. Name of the registry: researchregistry
2. Unique Identifying number or registration:6956
3. Hyperlink to your specific registration (must be publicly accessible and will be checked):

## Guarantor

Dounia sarfi.

## Declaration of competing interest

Authors of this article have no conflict or competing interests. All of the authors approved the final version of the manuscript.
